# Promoting Optimal Physical Exercise for Life: An Exercise and Self-Management Program to Encourage Participation in Physical Activity after Discharge from Stroke Rehabilitation—A Feasibility Study

**DOI:** 10.1155/2016/9476541

**Published:** 2016-05-30

**Authors:** Avril Mansfield, Svetlana Knorr, Vivien Poon, Elizabeth L. Inness, Laura Middleton, Louis Biasin, Karen Brunton, Jo-Anne Howe, Dina Brooks

**Affiliations:** ^1^Toronto Rehabilitation Institute, University Health Network, 550 University Avenue, Toronto, ON, Canada M5G 2A2; ^2^Department of Physical Therapy, University of Toronto, 500 University Avenue, Toronto, ON, Canada M5G 1V7; ^3^Evaluative Clinical Sciences, Hurvitz Brain Sciences Program, Sunnybrook Research Institute, 2075 Bayview Avenue, Toronto, ON, Canada M4N 3M5; ^4^Department of Kinesiology, University of Waterloo, 200 University Avenue W, Waterloo, ON, Canada N2L 3G1

## Abstract

People with stroke do not achieve adequate levels of physical exercise following discharge from rehabilitation. We developed a group exercise and self-management program (PROPEL), delivered during stroke rehabilitation, to promote uptake of physical activity after discharge. This study aimed to establish the feasibility of a larger study to evaluate the effect of this program on participation in self-directed physical activity. Participants with subacute stroke were recruited at discharge from one of three rehabilitation hospitals; one hospital offered the PROPEL program whereas the other two did not (comparison group; COMP). A high proportion (11/16) of eligible PROPEL program participants consented to the study. Fifteen COMP participants were also recruited. Compliance with wearing an accelerometer for 6 weeks continuously and completing physical activity questionnaires was high (>80%), whereas only 34% of daily heart rate data were available. Individuals who completed the PROPEL program seemed to have higher outcome expectations for exercise, fewer barriers to physical activity, and higher participation in physical activity than COMP participants (Hedge's *g* ≥ 0.5). The PROPEL program delivered during stroke rehabilitation shows promise for reducing barriers to exercise and increasing participation in physical activity after discharge. This study supports feasibility of a larger randomized trial to evaluate this program.

## 1. Introduction

Aerobic exercise is essential for stroke recovery [1]. People with stroke usually have diminished aerobic capacity [2], which makes even minimal activity effortful and, in conjunction with sensorimotor impairments, may restrict activities of daily living and independent function [2, 3]. Exercise can improve aerobic fitness poststroke [4]. Individuals with stroke who are physically active are more satisfied with their lives [5] and have improved quality of life [6] than those who are inactive. Aerobic exercise also improves cardiovascular health and is advocated for secondary stroke prevention [1]. Importantly, aerobic exercise is beneficial even early after stroke [7] and can be feasibly implemented during routine rehabilitation [8]. However, length of stay in stroke rehabilitation is relatively short, so ongoing exercise postdischarge is necessary to maintain these benefits.

Referral to other supervised exercise programs, such as adapted cardiac rehabilitation [9], could help people with chronic stroke to improve or maintain aerobic fitness. However, referral to such a program does not mean that a person will enroll (enrolment rates average 42% [10]) or actively participate (38% of people who enroll in cardiac rehabilitation attend less than half of the exercise sessions [11]). Additionally, participation in such programs is not sustainable indefinitely. Participation in self-directed physical activity is therefore necessary to maintain fitness in the long-term. However, studies consistently show that individuals with stroke are less active than age-matched controls. Individuals with stroke give up over half of the physical activities they engaged in prior to their stroke [5]. Physical activity declines after discharge from rehabilitation [12]. Community-dwelling ambulatory individuals with stroke typically walk fewer than 4,300 steps per day [13–16]. In a study where heart rate monitors were used to quantify the cardiovascular challenge of daily physical activity, none of the participants with stroke met the guidelines for frequency, intensity, and duration of physical activity [12]. Thus, even when individuals with stroke are physically active, the intensity of activity is not sufficient to bring about any changes in physical fitness [12, 17]. Chronic inactivity within this group means that any gains in aerobic fitness made during rehabilitation are quickly lost postdischarge [18]. Therefore, there is a need to establish strategies to promote long-term uptake of self-directed exercise after stroke [19].

Interventions to promote longer term self-directed exercise poststroke have been primarily directed towards patients once they return to the community after formal rehabilitation is complete [20, 21]. There may be an opportunity for targeted fitness programming during rehabilitation not only to increase aerobic capacity, but also to shape long-term self-directed physical activity behaviour upon return to the community [18]. Exercise self-efficacy predicts exercise behaviour after stroke [22–24]; therefore, it is plausible then that improving exercise self-efficacy prior to discharge from rehabilitation could influence long-term exercise behaviour. However, while fitness training during inpatient stroke rehabilitation is feasible and improves functional capacity [7, 8, 25], participation in supervised fitness training alone does not improve exercise self-efficacy [8] or increase physical activity following discharge into the community [26]. Group-based fitness programming with progressive self-management emphasizing goal-setting, problem-solving, and support during rehabilitation could be an appropriate strategy.

In our institution, we have implemented a program of care, Promoting Optimal Physical Exercise for Life (PROPEL), that aims to equip individuals after stroke with the knowledge, skills, and self-efficacy required to remain physically active following discharge from rehabilitation. The purpose of this study is to establish the feasibility of conducting a larger study to evaluate the effect of this program on participation in physical activity after the end of the program. To establish feasibility, we aimed to determine the rate of recruitment into and withdrawal from the study and rate of compliance with continuous activity monitoring for six weeks after completion of the program. Our secondary objective was to estimate the effect of the program on participation in physical activity (as measured with a questionnaire, accelerometer, and heart rate monitor), self-efficacy for exercise, outcome expectations for exercise, and barriers to being active, when compared to a group of participants who did not complete the program.

## 2. Methods

### 2.1. Participants

Individuals who completed the PROPEL program in- and outpatient modules (see below) at the Toronto Rehabilitation Institute, University Health Network, were invited to participate in this study. To be eligible for referral to the PROPEL program, patients must be admitted to the facility for rehabilitation services following a diagnosed stroke and must be able to understand instructions. Patients were excluded from the PROPEL program if they had uncontrolled hypertension, uncontrolled diabetes, other cardiovascular morbidities that would limit exercise tolerance (e.g., heart failure, abnormal blood pressure responses or ST-segment depression >2 mm, symptomatic aortic stenosis, or complex arrhythmias), unstable angina, orthostatic blood pressure decrease of >20 mmHg with symptoms, or musculoskeletal impairments or pain that would limit their ability to exercise. For the purpose of the current study, participants were excluded if they had a language or communication barrier that prevented completion of questionnaires (e.g., severe aphasia or non-English speaking), cognitive impairment that would prevent participation in unsupervised physical activity and/or if they lived >50 km from the research sites.

Patients who were enrolled in a longitudinal study of physical and cognitive recovery from stroke [27] and who had expressed interest in participating in other research studies were also invited to participate in this study to form a comparison (COMP) group. COMP participants received in- and outpatient rehabilitation at one of two hospitals that did not have a structured group aerobic exercise program available; the three hospitals were otherwise similar in terms of provision of rehabilitation services (i.e., ~1 hour per day, at least 5 (inpatient) or 2 (outpatient) days per week of each of physiotherapy, occupational therapy, and speech and language therapy, as needed). Regular physiotherapy sessions at all sites included strength, balance, and gait training, according to patients' individual needs. COMP participants met the same inclusion and exclusion criteria as PROPEL participants. PROPEL participants were enrolled into the study at the end of the outpatient module of the PROPEL program (see details below); COMP participants were enrolled approximately 6 weeks after discharge from inpatient rehabilitation such that the timing of data collection would align with that of the PROPEL group. During this 6-week delay, COMP participants either were enrolled in outpatient rehabilitation or were on a waiting list to start outpatient rehabilitation, which represents routine practice within our region. Therefore, PROPEL participants had also been attending outpatient rehabilitation or were on a waiting list for outpatient rehabilitation at the time of completing the PROPEL intervention.

The study was approved by the Toronto Rehabilitation Institute Research Ethics Board (TRI REB #: 12-037), and participants provided written informed consent prior to participation.

### 2.2. PROPEL Program

The PROPEL program is a supervised and individualized aerobic training group that is available three times per week, consisting of an inpatient module and an outpatient module. The inpatient module involved group exercise only in an open-group format, whereas the outpatient module involved group exercise in a closed-group format in addition to self-management components. The self-management components were reserved for the outpatient module as the closed-group format was considered essential to the intervention (see rationale below), but it would be challenging to arrange for closed-groups within inpatient rehabilitation where lengths of stay are variable between-patients and typically less than the 6-week duration of the self-management program. Additionally, completing self-management components after participants were discharged from inpatient rehabilitation and residing in the community meant that supportive planning and problem-solving to overcome barriers to exercise and achieve patient long-term goals of continued exercise would be fostered as patients transition from the fitness group to independent exercise in the community.

The inpatient module is described in detail elsewhere [8]. Patients were referred by their primary treating physiotherapist. An initial submaximal aerobic capacity test with electrocardiography was conducted on a recumbent stepper prior to entry into the program. The submaximal aerobic capacity test was stopped when patients reached 70% of age-predicted maximum heart rate (60% if taking beta-blockers), reported a rating of perceived exertion of 5 (out of 10), or were unable to maintain a constant stepping cadence. Patients were withdrawn from the program if cardiovascular abnormalities were observed during this test. Intensity and duration of training were determined by patients' physiotherapists based on the initial submaximal aerobic capacity test. The choice of exercise modality (e.g., recumbent stepper, cycle ergometer, or treadmill) was based on patients' sensorimotor recovery, postural control, functional abilities, and safety.

Group exercise was supervised by a physiotherapist and healthcare student. A typical exercise session included a 3–5-minute “warm-up” and “cool-down” of low-intensity exercise and 20–30 minutes of aerobic training at a target heart rate determined from the submaximal test on the prescribed exercise modality. Heart rate, blood pressure, rate of perceived exertion, workload, and duration of training were documented for each session. This log was reviewed by the physiotherapist on a regular basis with appropriate progression of the intensity and/or duration of exercise as necessary. Within the inpatient module, patients continued to exercise until discharge from inpatient rehabilitation.

For referral to the outpatient module, patients must be graduates of the inpatient module and must be a registered outpatient at Toronto Rehabilitation Institute. Ideally, the six-week outpatient module would begin immediately upon discharge from inpatient rehabilitation; however, patients were placed on a waiting list if there were not enough patients discharged at the same time to form a group. Components of the outpatient module were developed by integrating principles from the Transtheoretical Model [28] and Social Cognitive Theory [29] and, as such, have been designed to try to increase exercise self-efficacy. Successful performance of exercise within rehabilitation allows the experience of mastery, one of the most powerful methods to improve self-efficacy [30]. The group format allows vicarious experiences; seeing others' achievements, especially for those uncertain of their own capabilities, may improve beliefs in the individuals' own capabilities [30]. The group can also act as a motivator for continued engagement in physical activity to achieve personal goals [23, 31, 32] by offering encouragement to attempt new exercises and challenge negative perceptions of ability [32, 33]. Access to a healthcare professional leading the group can increase an individual's belief about personal skill [30], and teaching stroke survivors how to exercise independently can promote feelings of safety and confidence [33–37].

Within the outpatient module, patients continued to attend fitness classes up to three times per week and continued progressing with their aerobic training. Patients also attended small group sessions once weekly to learn self-management skills for exercise in preparation for discharge from rehabilitation. These small group sessions included discussions to identify and problem-solve barriers to exercise, to understand personal and general benefits of exercise, to explore appropriate community resources for exercise, and to find individualized and realistic strategies for incorporating exercise in a regular routine. The Stages of Change tool within the Transtheoretical Model was used to guide assessment of readiness to change. Based on an appreciation that participants presented at difference stages of readiness for change with respect to commitment to exercise, the program was designed to provide intervention that was aligned with participants' identified needs, what they wanted to learn or discuss, and what was relevant to them. With a goal of facilitating self-efficacy with this type of exercise, patients were shown how to monitor their own heart rate and rate of perceived exertion. Patients were also taught how to progress their aerobic exercise and were encouraged to set weekly exercise short-term goals, as well as longer term exercise goals. Caregivers were welcome to join the group discussions, if available and if participants wished to include them. We also encouraged participants to involve their caregivers, friends, and family when planning their next steps in sustaining their exercise program in the community (e.g., helping with transportation or using equipment). However, in keeping with our approach to tailor intervention to participant preferences, we respected participants' preferences to have caregiver involvement or to exercise independently. The aim of the outpatient module of the fitness group was to facilitate successful transition into appropriate and regular community or home-based exercise after discharge.

### 2.3. Measures

#### 2.3.1. Rates of Recruiting

This study took place between February 2013 and November 2014. During this time, we counted the number of individuals who were referred to the inpatient and outpatient modules of the PROPEL program. We also counted the number of individuals who were eligible for the study and documented reasons for ineligibility.

#### 2.3.2. Cohort Descriptors

The following information was obtained from clinical charts in order to characterize individuals who participated in the study: age, sex, time after stroke (at enrolment into the study), and the more affected side. Data collection for all other measures occurred in participants' homes. The National Institutes of Health Stroke Scale (NIH-SS) [38] and the Chedoke-McMaster Stroke Assessment (CMSA) [39] foot and leg scores were scored by a research assistant upon enrolment into the study. Premorbid exercise behaviour was evaluated using the Schmidt retrospective Physical Activity Scale [40]. We used this scale to estimate participants' average amount of time (hours/day) prior to their strokes spent in sedentary activities (e.g., watching television, sedentary occupational activity) and in physical recreational activity or exercise.

#### 2.3.3. Physical Activity

Physical activity during the 6 weeks following enrolment into the study (i.e., upon completion of PROPEL program or ~6 weeks after discharge from inpatient rehabilitation for the COMP group) was assessed using an accelerometer, heart rate monitor, and questionnaires. Physical activity measures primarily captured aerobic exercise due to the relative ease of obtaining objective measures of aerobic activity (i.e., walking from the accelerometer and heart rate data) and because there are clear guidelines regarding the recommended frequency and intensity of aerobic exercise [1, 41]. Other activities (e.g., balance and resistance training) might have been captured by the accelerometer and heart rate monitor, although the effort will likely be underestimated. However, the questionnaire also captured strength, balance, and flexibility exercises. Participants were supplied with an accelerometer and heart rate monitor (wGT3X+, ActiGraph, Pensacola, Florida, USA) at enrolment into the study. The accelerometer was worn around the waist (over or under clothing) using an elastic belt, and the heart rate monitor was worn under clothing around the chest such that the electrodes were in contact with the skin. The ActiGraph accelerometer worn at the waist shows good agreement with step counts in daily walking from a previously validated accelerometer among individuals with stroke (intraclass correlation coefficient (ICC) without filter: 0.82; ICC with low-frequency extension (LFE) filter: 0.90 [42]). Participants were instructed to wear both monitors for all waking hours over the six-week period following enrolment into the study. A research assistant visited participants in their homes every two weeks during the 6-week period to replace the devices so that data could be downloaded and the batteries could be recharged. At the end of each week, participants were asked to complete the Physical Activity Scale for Individuals with Physical Disabilities (PASIPD) [43]. This is a 13-item questionnaire in which participants are asked to indicate the frequency and duration of recreational, household, and occupational physical activities completed in the previous 7 days. The PASID has been validated within a group of individuals with various physical disabilities, including those with stroke, showing good test-retest reliability (*ρ* = 0.77) and moderate criterion validity (*ρ* = 0.30) when compared to accelerometer-based activity monitoring [44].

#### 2.3.4. Self-Efficacy, Outcome Expectations, and Barriers to Being Active

Exercise self-efficacy was assessed using the Short Self-Efficacy for Exercise (SSEE) scale [45]. The SSEE scale is a four-item questionnaire where participants are required to rate their confidence exercising through pain and fatigue and when alone and depressed on a five-point scale. The Short Outcome Expectation for Exercise (SOEE) scale [45] was used to assess beliefs and attitudes related to exercise. The SOEE scale is a five-item questionnaire where participants are asked to rate their beliefs regarding the benefits of exercise on a five-point scale. The SSEE and SOEE were assessed at enrolment into the study.

Perceived barriers to physical activity were assessed at the end of the 6-week monitoring period using the Barriers to Being Active Quiz (BBAQ) [15, 46]. Barriers were documented at the end, rather than the beginning, of this period as participants had more time to experience, and potentially solve, barriers to being active in the community. The BBAQ is a 21-item scale wherein individuals are required to indicate how likely they are to make specific statements regarding barriers to exercise: for example, “I'm getting older so exercise can be risky” [46]. Items on seven categories of barriers are included in the questionnaire: lack of time, social influence, lack of energy, lack of willpower, fear of injury, lack of skill, and lack of resources. Each individual item is scored from 0 to 3 and scores for each barrier category are the sum of the scores for the three items in that category. Participants are considered to have a “significant” barrier to being active if the score for a category is 5 or higher [15]. The average number of significant barriers per participant was calculated.

### 2.4. Data Processing and Analysis

Accelerometer and heart rate data were initially processed using ActiLife software (Version 6, ActiGraph, Pensacola, Florida, USA). To account for potentially underestimating walking activity among individuals who walk slowly, step counts were obtained both with and without the ActiGraph LFE [47]. Number of steps and average heart rate were calculated in 10-second epochs and were exported to a text file for further processing using a custom MATLAB routine (The Mathworks, Natick, Massachusetts, USA). Heart rate values that were considered to be physiologically improbable (<35 or >220 beats per minute) were removed. For each 10-second epoch, heart rate was considered to be within an appropriate target range for an aerobic benefit if it was 55–80% [1] of age-predicted maximum (208 − 0.7*∗*age [48]). The cumulative amount of time that heart rate was within this target range for at least 10 minutes continuously [1] was calculated for each day. Total number of steps taken per day was calculated. We assumed that the accelerometer was not worn on a given day if the total step count for that day was <10 steps and that the heart rate monitor was not worn if there were 0 minutes of physiologically probable heart rate values recorded for that day. The average number of days for which the accelerometer and heart rate monitor were worn was calculated for each participant. The average number of steps per day and the average time when heart rate was within the target range were calculated only for those days when the accelerometer or heart rate monitor was worn. We used the PASIPD questionnaire (items 4, 5, and 6: moderate sport and recreational activities, strenuous sport and recreational activities, and exercise to increase muscle strength or endurance) and heart rate data to determine if participants met recommendations for physical activity (>150 minutes per week of moderate and/or vigorous intensity activity [41]).

Baseline characteristics were compared between groups using Kruskal-Wallis tests (ordinal or ratio variables) or the chi-square test (categorical variables). Mean PASIPD scores, number of steps/day, and time when heart rate was within the target range were calculated over the 6-week period for each participant. Effect sizes for the between-group differences in PASIPD scores, number of steps/day, time when heart rate was within the target range, SSEE and SSOE scores, and number of significant barriers to physical activity were calculated as the raw difference in means and using Hedge's *g*.

## 3. Results

### 3.1. Referral and Recruiting Rates

Between February 2013 and November 2014, 125 patients were referred to the inpatient module of the PROPEL program; however, only 21 of these patients were referred to the outpatient module. There were 7 outpatient groups in this time, with a mode number of 3 patients per group. One patient was withdrawn from the outpatient module of the PROPEL program due to a change in health status, and two voluntarily withdrew. Of the remaining 18 patients, 2 were ineligible for this study due to foreign language, and 5 declined to participate. Therefore, 11 participants were enrolled in the study. Of note, two patients declined participation in the study because they thought the activity monitoring would be too difficult for them.

### 3.2. Participant Characteristics

The 11 study participants started the outpatient module of the PROPEL program, on average, 43.9 days after discharge from inpatient rehabilitation (range: 3–154 days). Participants were enrolled into the study on average 31.5 days after the end of the outpatient module (range: 0–82 days). Fifteen COMP participants were recruited. COMP participants were enrolled, on average, 45.5 days after discharge from inpatient rehabilitation (range: 22–96 days). Characteristics of participants at enrolment into the study are presented in Table 1. As noted above, due to delays in PROPEL participants starting the program and delays in starting the study after the end of the program, PROPEL group participants were enrolled much later after stroke than COMP group participants (167.5 days versus 94.2 days; *p* = 0.018). There were also significantly more men in the PROPEL group than the COMP group (*p* = 0.033), and PROPEL participants had higher CMSA-foot scores than COMP participants (*p* = 0.041). The two groups were comparable in terms of stroke severity, stage of motor recovery in the leg, and premorbid physical activity.

### 3.3. Compliance with Activity Measures

One PROPEL group participant completely withdrew from the study after three weeks. Two COMP group participants declined to wear the accelerometer and heart rate monitor after two weeks but were willing to complete the PASIPD and BBAQ. Data regarding compliance with activity monitoring are presented in Table 2. Participants in both groups were more compliant with completing the PASIPD questionnaires and wearing the accelerometer than they were with wearing the heart rate monitor; 96.7% of PASIPD scores, 80.5% of accelerometer data, and 34.3% of heart rate data were available. There were no valid heart rate data for 9 participants (3 PROPEL, 6 COMP).

### 3.4. Between-Group Comparison in Physical Activity Measures

**Figure 1:**
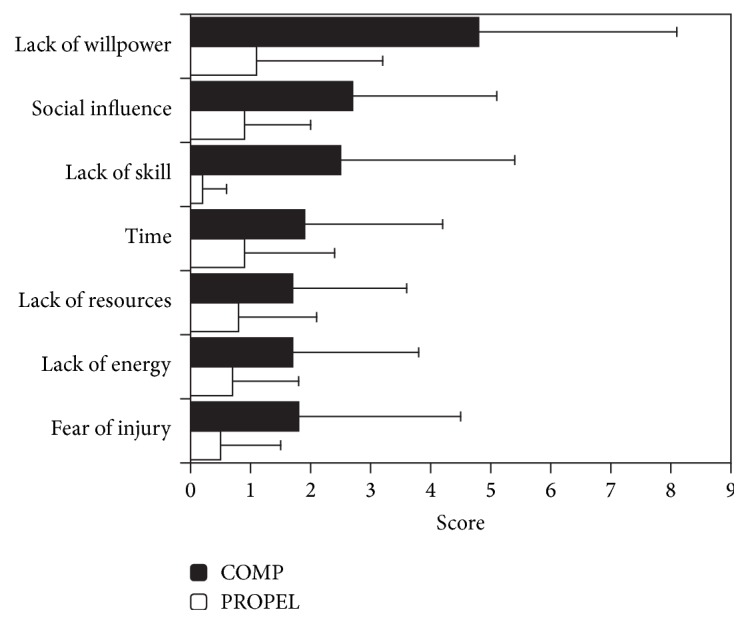
Category scores on the BBAQ. Values presented are the mean score for each category on the BBAQ, with standard deviation error bars.

Mean PASIPD scores, steps per day, time when heart rate was within the target range, SSOE and SSEE scores, and number of significant barriers to physical activity per group are presented in Table 3. PROPEL participants reported higher PASIPD scores than COMP participants (Hedge's *g*: 0.75). For at least half of the monitoring period, 8/11 PROPEL participants and 3/15 COMP participants (based on PASIPD scores) or 2/8 PROPEL participants and none (out of 9) of the COMP participants (based on heart rate data) met the recommended weekly duration and intensity of physical activity [41]. PROPEL participants also reported higher SSOE scores (Hedge's *g*: 0.50) and fewer significant barriers to physical activity than COMP participants (Hedge's *g*: −1.18). Indeed, only one PROPEL participant reported a significant barrier to physical activity (lack of willpower). Lack of willpower was the most prevalent barrier among COMP participants, with 10/15 reporting that this prevented them from being more physically active. The mean BBAQ scores for each category of barrier are presented in Figure 1; lack of willpower and social influence had the highest scores within both groups.

## 4. Discussion

This study aimed to establish the feasibility of a larger trial to determine the effect of the PROPEL program on physical activity after discharge from stroke rehabilitation. We identified several problems that would affect feasibility of such a larger trial. Rates of recruitment at the PROPEL site were extremely low, which was primarily because only 17% of individuals who were referred to the inpatient module were subsequently referred to the outpatient module. Individuals with significant communication difficulty (e.g., due to foreign language or aphasia) were unable to participate in group discussions and were, therefore, excluded from the outpatient module. Some individuals completed outpatient rehabilitation at other centres and others declined enrolment in the outpatient module due to lack of interest or transportation difficulties. We do not believe that low physical function was a major reason for nonreferral to the outpatient module as individuals with CMSA-foot and leg scores as low as 2 and 3, respectively, were referred [8]. However, we cannot be sure of specific reasons for nonreferral to the outpatient module as we did not collect these data, which is a limitation to the current study. Furthermore, as the outpatient module was delivered in a group format and referrals were low, participants were often placed on a waiting list until there were a sufficient number of individuals to form a new group. These logistical challenges meant that COMP and PROPEL groups were not comparable at baseline (e.g., PROPEL participants were recruited much later after stroke than COMP participants). These between-group differences at baseline could account, at least in part, for the observed between-group differences in physical activity. A randomized trial, whereby individual participants at one or more sites are randomized to the PROPEL program or a control group, would not be feasible for this particular intervention as this would leave too few individuals assigned to the intervention to create groups for the PROPEL outpatient module; the group format is essential to this intervention to allow for vicarious experiences [30]. Thus, a cluster randomized trial, whereby sites are randomized to either the PROPEL program or a control, may be necessary. Regardless of the study design, a future trial would need to ensure that participants in the intervention and control groups are recruited at a similar time after stroke and are comparable on baseline characteristics.

Notwithstanding the low rates of referral to the PROPEL outpatient module, a high proportion (16/21) of referred individuals were eligible for the study and a high proportion of these (11/16) consented to participate. These high rates of participation in the study suggest that recruiting for a future trial would be feasible provided that rates of referral to the program could be improved. Likewise, there was a low rate of attrition; all participants had some valid data. Compliance with completing the PASIPD questionnaires and wearing accelerometer for 6-weeks continuously was high. Participants were less compliant with wearing the heart rate monitor. The research assistant provided instructions to participants regarding proper placement of the accelerometer and chest strap during each home visit; however, she often observed that the devices were worn improperly despite repeated instructions. The chest strap may also have become dry during the day, leading to inaccurate measurements. Provision of daily reminders and instructions may have improved compliance with wearing the chest strap. Alternatively, compliance may have been higher if participants were only required to wear the heart rate monitor for 5 days at a time, as has been done in other studies [12]. While they are likely not as accurate as chest-strap based monitors [49], wrist- or arm-worn heart rate monitors are easier to wear and, therefore, might actually obtain more valid data than a chest-strap based monitor.

Activity levels were generally low in both groups. While other studies have suggested that individuals with stroke overestimate physical activity when evaluated with questionnaires [22], we believe this is unlikely in the current study as self-reported physical activity was very low. PASIPD scores were lower than scores of individuals who self-rate as “not active at all” [43], although scores in this previous paper are largely influenced by occupational physical activity; many of our participants were retired or on leave from work due to their recent strokes. PASIPD scores for both groups were similar to those of individuals discharged from our stroke rehabilitation hospital prior to implementation of the PROPEL program [26]. Despite the low levels of physical activity in both groups, we observed “medium” effects (effect  sizes ≥ 0.5) favouring the PROPEL group for PASIPD scores and heart rate time within target. Likewise, both PASIPD and heart rate data suggest that more PROPEL participants than COMP participants met recommendations for physical activity [41]. Previous studies have found that participation in supervised aerobic exercise program alone does not increase self-directed physical activity after discharge from inpatient stroke rehabilitation [26] or among community-dwelling stroke survivors [50]. Thus, the additional self-management component of the PROPEL outpatient module may have aided participants in addressing common barriers in order to increase physical activity. However, these results remain inconclusive due to the study design and the fact that the groups were not comparable at baseline; effectiveness of the program will have to be established in a larger trial.

Step counts, when calculated with the normal filter, were also low for both groups. On the PASIPD, many participants reported activities such as strength, balance, and flexibility exercise at home, stationary cycling, or swimming/aquafit. These types of activities might not be captured by the accelerometer, which is primarily designed to capture walking activity. Additionally, PASIPD scores reflect estimated energy expenditure of activities completed; however, individuals with stroke with low aerobic capacity might experience relatively high heart rates and, therefore, achieve an aerobic training benefit from activities with relatively low estimated energy expenditure. Inclusion of heart rate monitoring may provide more useful information about the relative intensity of physical activity and potential for aerobic benefit than questionnaires or accelerometers. Therefore, combining three methods of data collection (questionnaire, accelerometer, and heart rate monitor) can provide a more complete picture of daily exercise participation than relying on a single method [12, 15, 22].

Alternatively, step counts were much higher when the LFE was applied (mean > 7,000 steps/day for both groups). The LFE appears to improve the accuracy of step counts over short distances compared to the normal filter setting among individuals with stroke [51]. However, others have suggested that the LFE might overestimate step counts among individuals with Parkinson disease [47]. More specific guidelines regarding use of the LFE are, therefore, required.

PROPEL participants did not report higher self-efficacy for exercise on the SSEE compared to COMP participants. It is possible that the brief self-efficacy questionnaire used in this study is not sensitive enough to capture small changes. Alternatively, while self-efficacy for exercise is one of the most common and reliable predictors of exercise behaviour after stroke [22–24], a meta-analysis of behaviour-change interventions among obese individuals found that 19 studies reported increased participation in physical activity, whereas only 4 improved self-efficacy [52]. Additionally, others have suggested that there are two distinct constructs within exercise self-efficacy: ability to perform exercise tasks and confidence to exercise in spite of common impediments (e.g., feeling tired [53]). This latter form of self-efficacy is measured by the SSEE but only appears to improve following longer-duration interventions (>6 months [53]). Conversely, there is some evidence that task self-efficacy was high for PROPEL participants. In agreement with other studies “lack of skill” [15] was a prevalent barrier on the BBAQ for COMP participants; however, the “lack of skill” item received the lowest overall score for PROPEL participants. Therefore, the PROPEL program may have helped individuals to gain the knowledge and skills to be able to exercise independently. Future studies should evaluate different constructs of exercise self-efficacy. It was also encouraging that PROPEL participants reported greater outcome expectations for exercise and fewer barriers to physical activity than COMP participants (effects  sizes ≥ 0.5 for SSOE and number of significant barriers); indeed, only one PROPEL participant reported a significant barrier. In agreement with previous work [15, 54] lack of willpower was identified in the current study as the primary barrier to being move active in both groups.

## 5. Conclusions

This feasibility study emphasizes some design challenges for a future trial to evaluate the effect of a combined aerobic exercise and self-management intervention, delivered during rehabilitation, on participation in self-directed physical activity among individuals with stroke. While participants were not randomly allocated to the intervention and comparison groups and the groups differed on some key measures at baseline, participants who completed the self-management program seemed to report fewer barriers to physical activity and to participate in more physical activity than those who did not complete the program. The effectiveness of the program will need to be confirmed in a larger randomized controlled trial.

## Figures and Tables

**Table 1:** *Participant characteristics*. Values presented are means with standard deviations and ranges in parentheses. The *p* value is for the comparison between groups using Kruskal-Wallis test (continuous or ordinal data) or chi-square test (categorical data).

	PROPEL group (*n* = 11)	COMP group (*n* = 15)	*p* value
Age (years)	62.1 (10.0; 42–76)	67.1 (13.4; 46–87)	*0.29*
Sex (number)			
Men	9	6	*0.033*
Women	2	9	
Time after stroke (days)	167.5 (77.3; 66–283)	94.2 (25.3; 47–139)	*0.012*
More affected side (number)			
Left	5	7	*0.95*
Right	6	8	
NIH-SS (score)	2.4 (2.5; 0–7)	2.7 (1.9; 0–8)	*0.46*
CMSA-leg (score)	5.3 (1.3, 3–7)	4.7 (1.1, 3–7)	*0.20*
CMSA-foot (score)	5.0 (1.5, 2–7)	4.0 (1.0, 2–6)	*0.041*
Premorbid sedentary time (hours/day)	4.7 (2.3, 2.0–8.1)	4.3 (2.4, 1.4–7.7)	*0.42*
Premorbid exercise (hours/day)	0.15 (0.22, 0–0.53)	0.60 (0.83, 0–2.5)	*0.16*

CMSA: Chedoke-McMaster Stroke Assessment; NIH-SS: National Institutes of Health Stroke Scale.

**Table 2:** *Participant compliance with activity monitoring*. Values presented are the mean number of weeks or days per participant where valid data were collected, with standard deviation and range in parentheses, and mean percentage of valid data.

	PROPEL group	COMP group
PASIPD scores (weeks; max = 6)	5.6 (0.9; 3–6)	5.9 (0.5; 4–6)
PASIPD scores (%)	93	98
Accelerometer (days; max = 42)	33.1 (11.6; 1–42)	34.7 (8.2; 15–42)
Accelerometer (%)	79	83
Heart rate monitor (days; max = 42)	14.2 (14.1; 0–38)	14.8 (16.0; 0–42)
Heart rate monitor (%)	34	35

PASIPD: Physical Activity Scale for individuals with physical disabilities.

**Table 3:** *Effect sizes for main outcomes*. Values presented are means with standard deviations and ranges in parentheses. Effect sizes (Hedge's *g*) were calculated based on the difference between the PROPEL and COMP groups, divided by the pooled standard deviation.

	PROPEL group	COMP group	Difference	Hedge's *g*
PASIPD (score)	8.4 (3.1; 3.3–12.5)	5.9 (3.0; 1.4–10.9)	2.5	*0.75*
Steps without LFE (number/day)	2705 (1681; 215–5354)	2519 (2133; 251–7232)	185	*0.09*
Steps with LFE (number/day)^*∗*^	7499 (3303; 2364–12905)	7048 (2579; 3409–11517)	451	*0.14*
Heart rate within target (mins/day)^*∗∗*^	14.1 (20.3; 0–56.0)	4.0 (4.7; 0–13.2)	10.1	*0.63*
SSEE (score)	3.5 (1.1; 1.5–5.0)	3.5 (0.9; 1.5–5.0)	0.0	*0.00*
SOEE (score)	4.4 (0.4; 3.8–5.0)	3.9 (1.2; 1.0–5.0)	0.5	*0.50*
Significant barrier (number)	0.1 (0.3; 0–1)	1.7 (1.9; 0–6)	−1.6	−*1.18*

LFE: low frequency extension; PASIPD: Physical Activity Scale for Individuals with Physical Disabilities; SSEE: Short Self-Efficacy for Exercise, SOEE: short outcome expectations for exercise.

^*∗*^Steps with LFE based on *n* = 14 for the COMP group due to difficulty processing one participant's data with the LFE.

^*∗∗*^Heart rates within target times are based on *n* = 8 for the PROPEL group and *n* = 9 for the COMP group due to a large amount of missing data for this variable (see also Table 2).
